# Grafts for Ridge Preservation

**DOI:** 10.3390/jfb6030833

**Published:** 2015-08-07

**Authors:** Amal Jamjoom, Robert E. Cohen

**Affiliations:** Department of Periodontics, State University of New York at Buffalo, Buffalo, NY 14214, USA; E-Mail: rcohen@buffalo.edu

**Keywords:** alveolar ridge resorption, ridge preservation, augmentation, autografts, allografts, xenografts, alloplasts

## Abstract

Alveolar ridge bone resorption is a biologic phenomenon that occurs following tooth extraction and cannot be prevented. This paper reviews the vertical and horizontal ridge dimensional changes that are associated with tooth extraction. It also provides an overview of the advantages of ridge preservation as well as grafting materials. A Medline search among English language papers was performed in March 2015 using alveolar ridge preservation, ridge augmentation, and various graft types as search terms. Additional papers were considered following the preliminary review of the initial search that were relevant to alveolar ridge preservation. The literature suggests that ridge preservation methods and augmentation techniques are available to minimize and restore available bone. Numerous grafting materials, such as autografts, allografts, xenografts, and alloplasts, currently are used for ridge preservation. Other materials, such as growth factors, also can be used to enhance biologic outcome.

## 1. Introduction

Alveolar ridge resorption refers to the bone remodeling that occurs following tooth extraction. Araujo *et al.* [1] found that the coronal aspect of buccal bone was often comprised only of bundle bone and hypothesized that bone resorption would occur after tooth extraction. Other authors proposed that surgical trauma during extraction results in the separation of the periostium from the underlying bone, causing vascular damage and an acute inflammatory response, which mediates bone resorption [2,3]. In this review, we address bone resorption in human extraction sockets.

Alveolar ridge resorption following tooth extraction may lead to esthetic and functional defects. The defects can be so severe that implant placement can be difficult or impossible without using augmentation procedures [4,5]. Those defects also can interfere with the use of removable dentures. Leblebicioglu *et al*. [6] have shown that ridge height loss is greater in mandibular than maxillary sites, and ridge width loss is greater on the buccal plate in both the mandibular and maxillary sites. Thinner buccal plates also appear to be associated with more post-extraction resorption [6]. Other studies have shown that elevating a full mucoperiosteal flap may be associated with bone loss following tooth extraction [7], resulting in approximately 0.6 mm of crestal bone loss [3].

Following an extraction, bone resorption occurs in two phases. In the first phase, the bundle bone that anchors the tooth in the alveolar process through Sharpey’s fibers is rapidly resorbed and replaced with newly formed immature woven bone [8,9,10]. Woven bone then starts to be replaced with mature lamellar bone that fills with mature bone in about 180 days. In the second phase, the periosteal surface of the alveolar bone remodels through an interaction between osteoclastic resorption and osteoblastic formation, leading to an overall horizontal and vertical tissue contraction [10]. One aim of this review is to discuss the vertical and horizontal ridge dimensional changes and healing of the extraction sockets. It also provides an overview of the advantages of ridge preservation procedures and the grafting materials that can be used for this purpose.

## 2. Methods

An electronic search using the PubMed database of the U.S. National Library of Medicine, National Institutes of Health, was performed in March 2015. In addition, a manual search of the *Journal of Periodontology*, *Journal of Clinical Periodontology*, *The International Journal of Periodontics and Restorative Dentistry*, *The International Journal of Oral and Maxillofacial Implants*, and *Clinical Oral Implant Research* also was conducted. Key search words included alveolar ridge preservation, ridge augmentation, autografts, allografts, alloplasts, xenografts, and growth factors. All available publication years were searched, although papers published in the last 10 years were given greater consideration.

## 3. Dimensional Changes after Extraction

The vertical linear extent of alveolar bone resorption occurs primarily during the first 3–6 months following extraction [8,11]. The buccal plate of bone is the most affected because its crestal portion is comprised solely of bundle bone. It is also generally thinner than the lingual plate, about 0.8 mm at the anterior teeth and 1.1 mm at the premolar teeth [8].

In his re-entry study three months after extraction with no ridge preservation of maxillary anterior teeth [12], Aimetti observed a mean vertical reduction of 1.2 ± 0.8 mm at the buccal aspects of the edentulous ridge, a 0.9 ± 1.1 mm loss at the palatal aspects, and a 0.5 ± 0.9 mm loss at the interproximal sites, all determined with an acrylic stent. Using a parallel radiographic technique with an occlusal bite block, Moya-Villaescusa and Sanchez-Pérez [13] demonstrated no statistically significant differences in vertical dimensional change between single (4.16 mm loss) and multi-rooted teeth (4.48 mm loss) three months after extraction with no ridge preservation [8].

In three different re-entry studies six months after extraction with no ridge preservation (control groups) [14,15,16], measurements were made of the external and internal vertical bone loss of anterior or premolar teeth. External vertical bone loss measurements (e.g., the distance from the coronal aspect of a titanium pin embedded in the buccal plate to the coronal border of the buccal bony wall) were −0.86 ± 0.14 mm [14], −1.5 ± 0.26 mm [15], and −1.00 ± 2.25 mm [16], respectively. Internal vertical measurements (e.g., distance from the most apical end of the socket to the coronal border of the buccal bony wall) were −2.71 ± 0.89 mm [14], −3.94 ± 0.35 mm [15], and −4.00 ± 2.33 mm [16], respectively. In a consensus report by Hämmerle *et al.* in 2012 [17], mean vertical bone loss was reported to be 1.24 mm. In their controlled clinical trial, Barone *et al.* showed a vertical bone resorption of 1 ± 0.7 mm, 2.1 ± 0.6 mm, 1 ± 0.8 mm, and 2 ± 0.73 mm at the mesial, vestibular, distal, and lingual sites, respectively.

In the same re-entry studies, horizontal bone resorption was reported to be −4.43 ± 0.65 mm [14], 4.56 ± 0.33 mm [15], and −3.06 ± 2.41 mm [16]. In general, horizontal bone resorption is far greater than the mean vertical resorption over 3–7 months [8]. Similarly, Hämmerle *et al.* [17] reported a mean horizontal bone loss of 3.8 mm, and Barone *et al.* [18] showed a horizontal bone resorption of 3.6 ± 0.72 mm.

## 4. Extraction Socket Healing

Wound healing in the extraction sockets occurs through a number of processes, including hematoma and clotting, formation of granulation tissue, re-epithelialization, replacement of granulation tissue with connective tissue, and bone formation. In the first few minutes after tooth extraction, a blood clot consisting of erythrocytes and platelets that are trapped in a fibrous matrix forms within the extraction socket. Granulation tissue, a new connective tissue that is highly vascularized, then starts to form after 48 h and is completed by day seven. The granulation tissue is totally replaced by connective tissue in about 30 days. Concurrently, re-epithelialization starts after four days and is completed around six weeks, depending on the site of the extracted tooth. After six weeks, osteogenic cells from the apical aspects and the walls of the socket migrate into the developing granulation tissue, differentiate into mature osteoblasts, and initiate bone deposition that will be completed in 4–6 months [10,19].

## 5. Ridge Preservation

### Advantages

It is well established that post-extraction ridge preservation can be beneficial prior to implant placement [4,19].

Although one study has shown that ridge preservation does not completely prevent bone loss, it aids in reducing the extent of that loss [20]. In a systematic review, Vittorini *et al.* [21] concluded that ridge preservation has a slight advantage over no treatment due to less horizontal and vertical bone loss. In their meta-analysis [21], they noted that following tooth extraction, it is preferable to perform ridge preservation at esthetic areas where the buccal bone thickness is less than 1.5 to 2 mm when several teeth are extracted or when anatomical structures such as the maxillary sinus and mandibular canal are located in immediate proximity.

In a clinical and histological human study on maxillary and mandibular anterior teeth, Iasella *et al.* [22] found a significant difference in the horizontal alveolar ridge dimensional changes between extraction with no preservation (EXT) (decreased from 9.1 ± 1.0 mm to 6.4 ± 2.2 mm) and ridge preservation (RP) (decreased from 9.2 ± 1.2 mm to 8.0 ± 1.4 mm) using freeze-dried bone allograft and collagen membrane, favoring preservation (a difference of 1.6 mm). Also, a significant difference was observed in the vertical dimension. For the RP group, there was a gain of 1.3 ± 2.0 mm *vs.* a loss of 0.9 ± 1.6 mm for the EXT group (a difference of 2.2 mm). Barone *et al*.[18] found that an alveolar ridge preservation technique with collagenated porcine bone and a resorbable membrane was able to limit the vertical changes after tooth extraction. In that study, the control group showed vertical bone resorption of 1 ± 0.7 mm, 2.1 ± 0.6 mm, 1 ± 0.8 mm, and 2 ± 0.73 mm at the mesial, buccal, distal, and lingual sites, respectively, *vs.* 0.3 ± 0.76 mm, 1.1 ± 0.96 mm, 0.3 ± 0.85, and 0.9 ± 0.98 mm at the mesial, buccal, distal, and lingual sites in the test group, respectively. Also, ridge preservation demonstrated better efficacy in the horizontal dimension (−3.6 ± 0.72 in control *vs.* −1.6 ± 0.55 mm in test sites). Aimetti *et al.* [12] also found less vertical and horizontal changes when ridge preservation was performed using calcium sulfate hemihydrate than extraction with no preservation. Ultimately, the indications for ridge preservation include maintenance of the existing hard and soft tissues of the alveolar ridge, and to simplify subsequent treatment (such as implant or denture placement).

## 6. Materials Used for Ridge Preservation

A variety of materials are available for post-extraction ridge preservation. For optimal results, all grafts require an adequate blood supply, a form of mechanical support, and osteogenic cells supplied by the host, graft material, or both [23]. Graft materials should have some osteogenic, osteoinductive, or osteoconductive properties. Osteogenic grafts supply viable osteoblasts that form new bone. Osteoinductive grafts stimulate the host mesenchymal cells to differentiate into osteoblasts that eventually form new bone. Osteoconductive grafts act as a scaffold or lattice for the surrounding cells to infiltrate and migrate through the graft.

### 6.1. Autogenous Bone

Autogenous bone is transferred from one position to another within the same individual. Autografts are biocompatible and have the potential to form new bone through osteogenesis, osteoinduction, and osteoconduction [24,25,26,27,28] However, autogenous bone grafts present several disadvantages, such as a limited amount of material, donor site morbidity, unpredictable bone quality, and post-operative discomfort [26]. Autogenous grafts can be cortical, cancellous, or cortico-cancellous. Cancellous autogenous bone is generally preferred, as it is rapidly re-vascularized and integrated into the acceptor site [27]. Cortical bone autografts are associated with a greater rate of apposition replacement and bone matrix resorption that can form foci of necrotic tissue [27]. Autogenous bone can be obtained from intra-oral or extra-oral sites and can be used in block or particulate forms [28]. Potential intra-oral donor sites frequently include the maxillary tuberosity, edentulous ridges, and exostoses for particulate autografts, while the chin and rami are common for block grafts [28]. Autogenous bone can be used alone or combined with other bone substitutes to form composite grafts [12,17]. Potential extra-oral sites include the iliac crest (most common), rib, and tibia. However, harvesting the bone from extra-oral sites has several disadvantages, including the need for hospitalization, prolonged recovery times, and graft sequestration [17].

### 6.2. Bone Substitutes

Several types of bone substitutes are commercially available, including allografts (from genetically similar members of the same species), xenografts (from other species), and alloplasts (of synthetic origin) [20,29,30]. Bone substitutes ideally should be able to form new bone and be biocompatible, completely resorbable, non-antigenic, non-carcinogenic, inexpensive, and pose no risk of disease transmission. They should also be space-maintaining, and have a similar composition, particle size, and resorption rate as human bone [23,31].

#### 6.2.1. Allografts

Allografts can be fresh-frozen, freeze-dried, or demineralized freeze-dried. The use of freeze-dried bone allografts (FDBA) and demineralized freeze-dried bone allografts (DFDBA) has reduced the problem of immunogenicity that was associated with fresh-frozen bone. They are the most common allografts used currently for ridge preservation [28].

FDBA revascularization occurs through integration/replacement (creeping substitution) at the recipient site and the formation of connective tissue areas. Small particles of the allograft may remain for several months to a year before they are completely resorbed [27,28]. Whittaker *et al.* [32] showed that allografts have both osteoinductive and osteoconductive properties, while other studies [33] claimed that allografts have only osteoinductive properties. Al-Ghamdi *et al.* [28] suggested that FDBA is only osteoconductive, while DFDBA can be both osteoconductive and osteoinductive. DFDBA also showed more vital bone and less residual grafting material compared to FDBA when placed in extraction sockets 19 weeks after extraction [34]. Studies comparing cortical and cancellous FDBA demonstrated no significant differences in the percentage of new bone formation at extraction sites [35].

The extent of allograft osteoinductivity depends on the donor age and the amount of bone morphogenetic proteins (BMPs) present in the graft. Grafts obtained from younger donors generally have more BMPs and are more osteoinductive [28]. FDBA and DFDBA have been widely used for regenerative therapy and ridge preservation [36]. In a histological study [36], Yukna and Vastardis compared bone regeneration with FDBA or DFDBA and noted more regeneration with FDBA. Dahlin [37] also showed that the reconstruction of atrophic maxillae with DFDBA, combined with guided bone regeneration (GBR technique), could be performed with similar treatment outcomes to autologous bone obtained from the iliac crest.

To avoid disease transmission from allografts, several chemical and physical processing techniques have been used. Chemical treatment with agents, such as 5% peracetic acid, 0.1% ethylenediaminetetraacetic acid, or 0.1% sodium dodecylsulfate, can alter the bone structure but may not sufficiently inactivate pathogens. Physical treatment, such as ultrasonication, may alter the microcrystal structure of bone mineral and denature organic components. With FDBA and DFDBA, more satisfactory results have been obtained through lyophilization, but cellular debris might remain after this treatment that could interfere with healing [28]. Tutoplast™ processing uses a multi-step preservation and sterilization process to remove tissue antigenic properties and is reported to inactivate pathogens without changing the structure, biomechanics, and convertibility of the tissues [28].

#### 6.2.2. Xenografts

Xenografts are derived from a variety of sources, including bovine, porcine, equine, and coralline, and are generally biocompatible and structurally similar to human bone [38]. Xenografts are osteoconductive and less frequently associated with the formation of interposition areas of connective tissue, but are not osteoinductive in humans [38].

Bovine xenografts are the most commonly used [39]. They contain similar hydroxyapatite content to that of human bone, which allows the graft to revascularize and be replaced by new human bone [38]. Xenografts originally were used to treat periodontal infrabony defects and generally resulted in new attachment and cementum formation when compared to ungrafted sites [38]. Bovine bone is associated with a 20%–40% retention of the graft after six months, as well as after three years, following placement [39]. The slow substitution rate allows long-term space maintenance. Other histological studies show good integration of bovine xenograft particles with newly formed bone filling the interparticulate space, forming direct contacts with the grafting material [23]. Methods to reduce antigenicity are similar to those used to process allografts [27].

Kotsakis *et al.* [40] compared the primary stability of implants placed in extraction sockets receiving either anorganic bovine bone (BOV) (*n* = 12) or calcium phosphosilicate putty (PUT) (*n* = 12). They showed that PUT can be more suitable for achieving primary stability for implants placed 5–6 months after ridge preservation.

Heterologous equine bone (DEB) (OX^®^, OsteoXenon^®^, Vicenza, Italy) in block or particulate forms has recently been introduced [41]. With DEB, antigenic materials are enzymatically degraded at low temperatures (<37 °C) to preserve type I collagen. This might allow enhanced bone regeneration, as type I native collagen activates both osteoblast and osteoclast adhesion and differentiation, as well as growth factor release [42]. In addition, the collagen gives the material an elasticity that makes it easier to shape and fit into a defect.

One study showed that DEB was less than ideal for crestal bone reconstruction [33]. In this study, there was a very high complication rate using both block and particulate forms. Block grafts had a greater than 50% failure rate in the immediate post-operative period, while GBR and sinus augmentation had a greater than 25% rate of infections and resorption, as well as late failures (after-implant placement). In a case series, other authors [42] showed comparable bone regenerative results at six months for bovine- and equine-derived xenografts for maxillary sinus augmentation.

#### 6.2.3. Alloplasts

Alloplasts are synthetic bone substitutes that act as a biologic filler with limited periodontal regeneration when used in treating periodontal bone defects [38]. They are osteoconductive bone substitutes [23,38]. As they are completely synthetic, they do not require a donor site, are available in unlimited quantities, and do not pose a risk of disease transmission [23,38]. The manufacturer also controls the particle size and interparticulate spaces, and typically makes them resemble natural bone [23].

The original alloplast material was Plaster of Paris, which is non-inflammatory, nonreactive, and encouraged bone healing in a contained lesion. All of the bio-ceramics are non-immunogenic, and are available in an unlimited supply [43].

Synthetic hydroxyapatite can be manufactured in different forms, including non-porous non-resorbable, dense non-resorbable, resorbable, porous, or non-ceramic forms [38]. Hydroxyapatite resorbs slowly over a period of years, and can be used for long-lasting ridge preservation [38].

Tricalcium phosphate, or TCP [Ca_3_(PO_4_)_2_], is a porous, osteoconductive grafting material. TCP is treated with naphthalene and then compacted at 1100–1300 °C to obtain a porosity diameter of 100–300 μm. During reabsorption, it supplies calcium and magnesium ions and creates an ionic environment similar to human bone. The ionic environment induces alkaline phosphatase activation, which is fundamental for bone synthesis [39]. TCP occurs as α and β phases. Although both phases have excellent resorbability and are chemically identical, they behave differently in a biologic environment. β-TCP transforms automatically but irreversibly into a more resorbable α-form at 1160 ± 40 °C. However, β-TCP is usually preferred as a biomaterial for its chemical stability, mechanical strength, and bioresorption properties [44]. β-TCP has been shown to be biocompatible and osteoconductive in both animal and clinical studies [31]. One study has shown unpredictable results with β-TCP because the particles become encapsulated with fibrous tissue [38]. Other studies have suggested that because β-TCP resorbs so quickly, it loses its space-making capacity. To address this problem, a biphasic calcium phosphate has been developed, consisting of a homogenous 60/40 mixture of hydroxyapatite (HA) and β-TCP. β-TCP will dissolve, providing calcium as well as space for bone formation. Meanwhile, more slowly resorbing HA maintains the scaffold [23].

Commercially available medical grade calcium sulfate hemihydrate (CSH) is the result of the partial dehydration of gypsum, which produces calcium sulfate hemihydrate (CaSO_4_.1/2H_2_O). Two forms are obtained depending on the method of calcination, α-hemihydrate or β-hemihydrate. β-hemihydrate is a fibrous aggregate of fine crystals with capillary pores, while α-hemihydrate consists of cleavage fragments and crystals in the form of rods or prisms. The product obtained from mixing the α-hemihydrate with water is stronger and harder than that resulting from the β-hemihydrate [45]. When implanted, it dissolves into calcium and sulfate ions. Calcium ions then combine with phosphate ions from body fluids to form calcium phosphate. Calcium phosphate is an osseo-conductive apatite [30] that stimulates bone ingrowth into the defect. The newly deposited material is similar to the apatite naturally present in bone. Calcium sulfate hemihydrate is biocompatible, biodegradable, safe, and non-toxic [43,45]. It also was shown that calcium sulfate has angiogenic and hemostatic properties. It has been used in bone defects secondary to trauma or tumors [46], post-extraction sites [29,47], maxillary sinus augmentation [48], periodontal infrabony defects [49], and as a barrier in guided tissue regeneration around implants [50].

Bioactive glass is an osteoconductive bone substitute composed of sodium oxide, calcium oxide, phosphorus pentoxide, silicon dioxide, and silica. The formation of a biologically active hydrated calcium phosphate layer at the surface of the bioactive glass plays a key role in the formation of the bone/graft bond [51].

### 6.3. Growth Factors

The incorporation of growth factors during regenerative therapy provides the opportunity to accelerate new bone formation and ridge preservation [23]. Growth factors are the signaling molecules that modulate cell growth and development. They play a role in cell proliferation, migration, and extracellular matrix formation. Some of the most important growth factors involved in bone homeostasis include platelet-derived growth factor (PDGF), transforming growth factor-β, fibroblast growth factor, insulin-like growth factor, vascular endothelial growth factor, parathyroid hormone, and bone morphogenetic proteins (BMPs) [23].

Two of the more extensively studied growth factors are PDGF and the BMPs, which are osteoinductive. They can be added to allografts, xenografts, or alloplasts to convert them from osteoconductive into osteoinductive materials by stimulating undifferentiated mesenchymal cells to differentiate into osteoblasts that form new bone [23,52,53]. Those growth factors can be semi-purified natural materials, such as platelet-rich plasma (PRP), platelet-rich fibrin (PRF), and enamel matrix proteins. They also can be recombinant human proteins, such as bone morphogenetic proteins (BMPs). The manufacturing of growth factors eliminates the issue of different concentrations of growth factors found in natural materials. It also allows for the use of a single protein at any concentration needed. Accordingly, BMP-2 has been developed as a recombinant growth factor and currently is used in periodontics for ridge preservation after extraction [54] and maxillary sinus augmentation [55]. In a systematic review, de Freitas *et al.* [55] concluded that recombinant human BMP-2 with an absorbable collagen sponge (ACS) carrier could be used as an alternative to autogenous bone grafts for alveolar ridge/maxillary sinus augmentation.

Enamel matrix proteins (Emdogain (EMD), Institut Straumann AG, Basel, Switzerland) are growth factors that are extracted from the tooth buds of piglets and suspended in a polyglycol gel. EMD contains over 95% amelogenin, with the remainder consisting of enamelin and other proteins [23,56]. One study showed histological evidence of regeneration in experimentally created periodontal defects in primate models [57], while others showed that EMD may be useful for treating human angular periodontal defects when combined with a modified Widman flap procedure [58]. Further studies have shown that EMD also stimulates production of further growth factors, such as BMPs [56].

PRP has three- to four-fold higher concentrations of platelets than a whole blood platelet count. It is prepared from autologous blood with a gradient density cell separator. When activated by thrombin or collagen, platelets can release the content of their granules to initiate coagulation cascade events. The granules release factors such as platelet-derived growth factor, transforming growth factor β, fibrinogen, vascular endothelial growth factor, fibronectin, von Willebrand factor, and P-selectin [45]. Currently, PRP has become a valuable adjunct to promote healing in mandibular reconstruction, surgical repair of the alveolar cleft, treatment of infrabony periodontal defects, and periodontal plastic surgery [59].

The use of PRF during bone grafting offers numerous advantages. First, the fibrin clot can maintain and stabilize the graft. Second, the fibrin network integrates into the regenerative site and facilitates cellular migration, particularly for endothelial cells necessary for angiogenesis, vascularization, and survival of the graft. It also helps in soft tissue maturation. During healing, platelet cytokines (PDGF, TGF-β, IGF-1) are released and participate in the healing process. Finally, the presence of leukocytes and cytokines in the fibrin network regulates the inflammatory and infectious phenomena associated with grafting [60,61,62].

PRF has been assessed in several other studies for its effect on healing and bone regeneration. PRF was associated with early healing of soft tissue overlying extraction sites after the first four weeks. After the first week, horizontal resorption on buccal aspects of extraction sites (1.07 ± 0.31 mm) was significantly less than that of the ungrafted sites (1.81 ± 0.88 mm) [63]. Animal studies using combination therapy with PRF showed accelerated healing in a rabbit calvarial defect model compared to unfilled defects [64]. Moreover, the restoration of peri-implant defects in rabbit tibia using PRF in combination with silk fibroin powder also showed promising results [64]. In two studies [65,66], PRF was used as an effective sole-filling material during simultaneous sinus lift and implant placement. It also proved to be an effective modality of therapy in the regenerative periodontal treatment of class II mandibular furcations [67].

Other tissue engineering approaches include cell culture to create cell sheets from fibroblasts or scaffolds rich in cells that can form membranes, as well as the use of stem cells and immortalized dental follicle cells, for periodontal regeneration [23].

### 6.4. Composite Grafts (Bone Substitutes Plus Growth Factors)

Combining tricalcium phosphate with growth factors, such as platelet-derived growth factors (PDGF), enhances osteogenesis [68]. Studies have shown that the combination therapy of PRP and CSH may lead to greater vital bone volume and more rapid bone healing after three months when compared to sites with a resorbable collagen dressing (RCD) [43,69]. Ridge augmentation and sinus grafts combining FDBA and PRP also have been shown to provide a viable therapeutic approach for implant placement [70]. When comparing sinus augmentation using FDBA and PRP *vs.* FDBA and resorbable cross-linked collagen membranes, Kassolis *et al.* [70,71] found a significant difference in the rate of new bone formation with the former approach.

### 6.5. Barrier Membranes

One study has shown that barrier membranes minimize alveolar bone resorption regardless of the use of additional grafting material [10]. Barrier membranes can be non-resorbable, such as expanded polytetrafluoroethylene (ePTFE) and titanium [19], or resorbable, such as polypeptides (collagen) and synthetic polymers (polylactide and polyglycolide).

Titanium is a highly reactive metal that gives rise to surface oxides that protect it from further degradation. The surface oxides have osteoconductive properties [72]. The rigidity of titanium membranes allows for greater stability in maintaining their role as space makers. The primary drawback associated with using non-resorbable membranes alone is that they generally require primary soft tissue closure. This positions the mucogingival junction more coronally, thus decreasing the width of keratinized tissue. It also may increase post-operative swelling and discomfort [73]. Furthermore, if the membrane becomes exposed, there is an increased risk of graft infection, which will affect regeneration [73].

The need to limit the exposure of non-resorbable membranes has led to the formulation of resorbable membranes (collagen, polylactide, polyglycolide) that are degraded in the human body. Those materials have been shown to be more biocompatible and flexible, thereby reducing membrane exposure. Non-cross-linked membranes rapidly resorb within weeks or months. Membrane availability can be prolonged by using a double layer or by collagen cross-linking, which may be advantageous when used in conjunction with grafts that have a low substitution rate. Studies in rat models have demonstrated decreased tissue integration and fewer blood vessels using cross-linked collagen membranes.

Resorbable collagen membranes, which are primarily derived from bovine sources, are composed of collagen types I and III. Membranes derived from porcine collagen also have been used (Bio-Gide, Geistlich, Wolhusen, Switzerland). Collagen membranes require a graft material to support them.

Resorbable membranes also include acellular dermal matrix grafts (ADMG; Alloderm, Biohorizons, Birmingham, AL, USA). Obtained from human skin, it is processed to remove the epidermis and cellular components, which eliminates the risk of rejection and reduces the risk of disease transmission. The material is very similar to collagen in structure.

### 6.6. Other Grafting Materials

One study [74] assessed the value of using RCD (CollaPlug) over the bone graft to protect the graft, induce hemostasis by stabilizing the blood clot, stimulate platelet aggregation, enhance fibrin linkage, and attract fibroblasts. Another author also reported the use of a polylactide and polyglycolide sponge as a space filler following extraction [75]. Their results indicated that alveolar bone resorption may be prevented or reduced by the use of the bioabsorbable synthetic sponge. Also, the bone that formed at six months was mineralized, mature, well-structured with no residual material detected, and was suitable for dental implant insertion.

## 7. Conclusions

Alveolar ridge resorption following tooth extraction may lead to esthetic and functional defects. The defects can be so severe that restorative dentistry can be difficult or impossible without using augmentation procedures [4,5]. Significant dimensional changes generally occur following extraction, but ridge preservation procedures have the potential to limit the extent of horizontal and vertical ridge alterations and preserves treatment options, particularly in the case of implant dentistry, where sufficient bone support is critical. Nevertheless, there is no single technique that appears to be superior, and the choice of materials may depend on the individual clinical situation and the restorative treatment plan.
